# Live-single-cell phenotypic cancer biomarkers-future role in precision oncology?

**DOI:** 10.1038/s41698-017-0025-y

**Published:** 2017-06-15

**Authors:** Grannum R. Sant, Kevin B. Knopf, David M. Albala

**Affiliations:** 1 Department of Urology, Tufts University School of Medicine, 82 Dennison Street, Gloucester, MA 01930 UK; 2 Cancer Commons, 35050 El Camino Real, Los Altos, CA 94022 USA; 3 Department of Urology, Crouse Hospital, Syracuse, NY USA

## Abstract

The promise of precision and personalized medicine is rooted in accurate, highly sensitive, and specific disease biomarkers. This is particularly true for cancer-a disease characterized by marked tumor heterogeneity and diverse molecular signatures. Although thousands of biomarkers have been described, only a very small number have been successfully translated into clinical use. Undoubtedly, there is need for rapid, quantitative, and more cost effective biomarkers for tumor diagnosis and prognosis, to allow for better risk stratification and aid clinicians in making personalized treatment decisions. This is particularly true for cancers where specific biomarkers are either not available (e.g., renal cell carcinoma) or where current biomarkers tend to classify individuals into broad risk categories unable to accurately assess individual tumor aggressiveness and adverse pathology potential (e.g., prostate cancer), thereby leading to problems of over-diagnosis and over-treatment of indolent cancer and under-treatment of aggressive cancer. This perspective highlights an emerging class of cancer biomarkers-live-single-cell phenotypic biomarkers, as compared to genomic biomarkers, and their potential application for cancer diagnosis, risk-stratification, and prognosis.

## Introduction

Successful and meaningful biomarkers can be applied to different touch points in the diagnosis and treatment of disease. Broadly, successful biomarkers can be classified into the following categories: screening–is the patient at risk of a disease; diagnostic–does the patient have a disease; prognostic–how aggressive is the patient’s disease; theranostic/companion diagnostic–which therapy or procedure will best cure or mitigate morbidity and mortality of a patient’s disease; and monitoring–has the disease been treated effectively, recurred or spread. Live cell biomarkers are poised to be a powerful addition to available biomarkers and can be assessed either via slice cultures,^1^ organoid cultures,^2^ or single cell cultures.^3^ In the following we discuss the application of live-single-cell phenotypic biomarkers across the continuum of patient diagnosis and treatment.

**Fig. 1:**
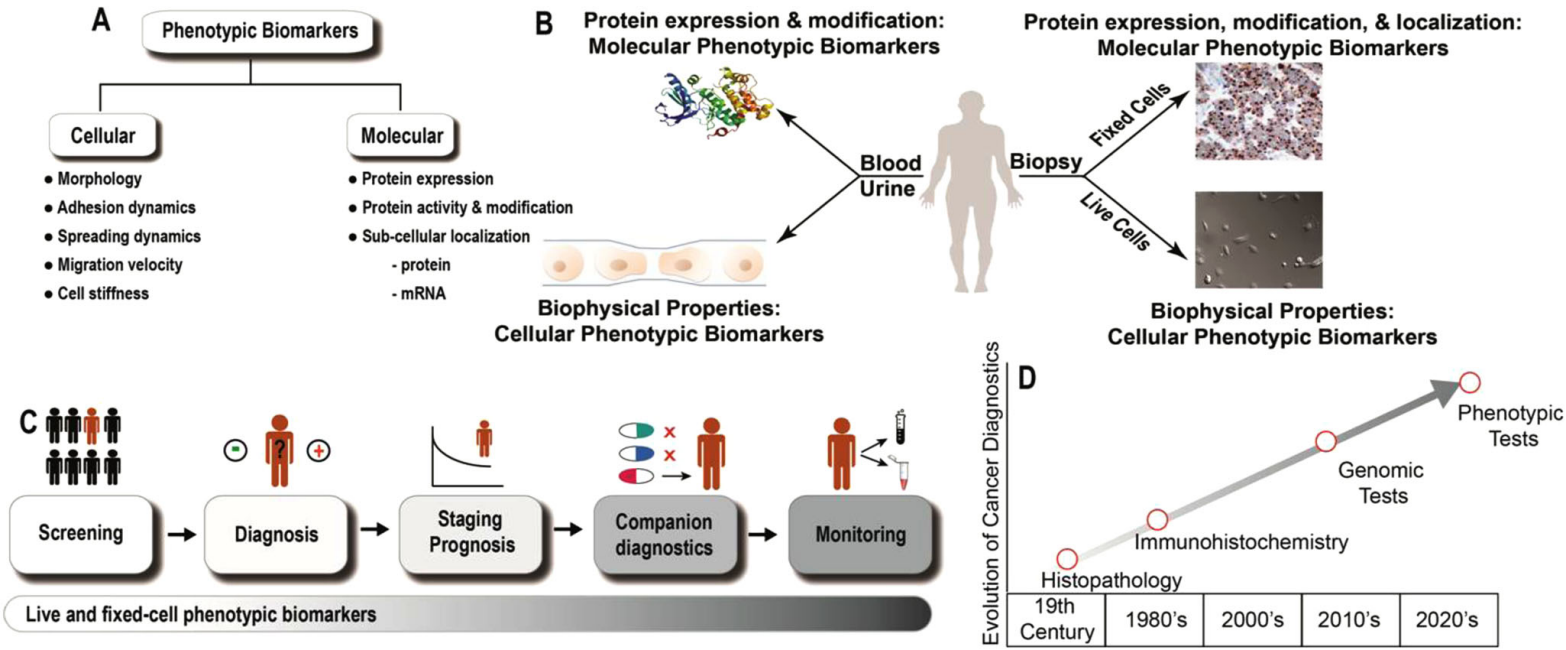
An overview of phenotypic biomarkers in cancer. **a**. Revised definition of phenotypic biomarkers: There are two sub-classes of phenotypic biomarkers. Cellular phenotypic biomarkers include non-molecular characteristics of cells such as morphology, adhesion dynamics, spreading dynamics, migration velocity, stiffness, and other biophysical parameters, which can be measured in live-cells. Molecular phenotypic biomarkers include proteins (expression, 10 activity and subcellular localization) and mRNA localization, which can be measured in fixed-tissue or cells. **b**. Sources of phenotypic biomarkers for clinical use: Patient biopsy samples can either be immunostained to evaluate molecular phenotypic biomarkers or be used to harvest and culture live-cells to evaluate cellular phenotypic biomarkers. Each individual cell can be further evaluated for molecular phenotypic biomarkers. Body fluids (blood/urine) are other sources of molecular phenotypic 15 biomarkers. **c**. Clinical applications of phenotypic biomarkers in cancer management: Phenotypic biomarkers are useful for screening (to identify at-risk individuals); diagnosis (to definitively detect presence of disease); staging and prognosis (to risk stratify and predict disease outcome); companion diagnostics (to predict response to drugs) and for monitoring disease (to evaluate therapeutic response and recurrence). **d**. Past, present and future of precision medicine

Similar to cardiovascular, metabolic, neurological, and other disease states, cancer is understood as being a complex disease with dynamic genetic and environmental risk factors in need of personalized biomarkers to triage disease susceptibility, occurrence, risk, treatment, and progression (Fig. 1). Cancer biomarkers have been broadly classified as either genomic (DNA/RNA) or phenotypic (morphology and protein expression). Classically, biomarkers have been measured using fixed tissue, yielding static biomarker results such as morphology, size, nuclear-cytoplasmic ratio, and genomic sequence information.^4, 5^ Importantly, these static biomarkers were observed in the context of formalin-fixed tissue samples that do not lend themselves to live-cell single cell analysis.^5^ Technological advances have improved the ability to define cellular phenotypes, and this previous narrow definition of phenotypic biomarkers to only include cell shape and proteins, is now incomplete. Analyses of live single-cell behavior suggest that dynamic biophysical cellular properties characterize both normal and disease states and represent a powerful extension and expansion of the concept of phenotypic biomarkers.^6^ Thus, a working definition of phenotypic biomarkers may be expanded to consider spatial and temporal considerations, including two phenotypic biomarker sub-categories: molecular and cellular (Fig. 1a) either measured via live-cell microscopy or fixed-cell microscopy.

Molecular phenotypic biomarkers include biomolecular parameters like the expression, activity and subcellular localization of proteins and mRNA. Cellular phenotypic biomarkers include morphology, as well as live-cell biophysical parameters such as cell motility, contractility, force generation, and cytoskeletal dynamics. Molecular biomarkers assess discrete components of multiple specific biochemical pathways whereas cellular biomarkers provide an integrated readout of several underlying biochemical and biological processes. Figure 1b lists the main phenotypic biomarkers that can be measured in body fluids (blood/urine) or biopsy tissue derived material, to provide information at either the tissue or single cell level. Fixed-cell phenotypic biomarkers such as cell morphology and tissue architecture are widely accepted as markers of disease, and will not be discussed here. Below, we summarize select cellular and molecular phenotypic biomarkers that have the potential to be translated and adopted into personalized novel quantitative and actionable cancer diagnostics and prognostics.

## Molecular phenotypic biomarkers

Molecular phenotypic biomarkers as defined as the expression, localization, dynamics, and modification state of DNA, RNA, and proteins can be measured in fixed or living tissue at the bulk-tissue level or single-cell level and can be derived from either tissue or bodily fluids. Further, molecular phenotypic biomarkers can be observed via different modalities such as, but not limited to, next generation sequencing, immuno-histochemistry (IHC), live-cell imaging, and functional magnetic resonance imaging. Each imaging modality, and related modalities employ different probes with their own binding kinetics that dictate the sensitivity and specificity of their respective temporal and spatial resolutions, engendering different ideal applications such as screening, diagnostics, prognostics, theranostics (or companion diagnostics), and monitoring, as well as potentially predicating their success in a given application.

With that, live-cell phenotypic biomarkers represent an un-tapped wealth of biomarkers due to difficulty around keeping biopsy tissue a live in vitro, as well as automated imaging and big-data/high-content data analysis techniques.^7, 8^ Given recent advances in our understanding of the tumor environment, microfluidics, high-content screening coupled with machine vision and big-data techniques (such as machine learning) it is conceivable to postulate a paradigm shift in the way patient tissue samples are analyzed^9^ with a transition of qualitatively assessing static, fixed tissue towards a modality of quantitatively analyzing live tissue. Further, the investigation of dynamic live-cell phenotypic biomarkers either in native micro-extracellular environments or specifically engineered extra-cellular environments may allow the level of interrogation, dissection and delineation of complex disease signaling pathways towards major clinical and therapeutic advances. As an example, probing cell-extracellular matrix (ECM) interactions or the manner in which cells interpret the adjacent microenvironment, is emerging as another ‘–omic’-like strategy to assess disease.^10^

Specifically, integrins, the primary mechano-transducers between cells and the ECM, recruit proteins to sites of cell–ECM contacts known as focal adhesion complexes.^11^ Integrins and focal adhesion proteins promote cellular growth and motility and are implicated in the ontogeny of epithelial tumors including prostate cancer.^12^ Focal adhesion protein complexes are coupled with the forward (protrusive) and rearward (retrograde) movement of actin.^13^ This interaction in turn regulates cell motility, growth, and proliferation.^14, 15^ Thus, the number and size of focal adhesions are indicative of malignancy and metastasis, and are emerging to be informative as molecular phenotypic tumor biomarkers.^16–18^ Several studies have reported a strong association between cancer progression and aberrant expression/activity of proteins involved in cell-cell adhesion, such as Cadherins and Catenins.^19, 20^ Morgan et al. report that abnormal expression of α and β Catenin, and Claudin 7 could serve as biomarkers to distinguish localized from metastatic prostate cancer.^20^ Broadly, as human cells transform from being benign to malignant, they demonstrate an increasingly disorganized cytoskeleton, decreased peripheral cellular actin and changes in cell adhesion properties.^18, 21, 22^ The concentrations and molecular architecture of various cytoskeletal components determine the overall mechanical response of cancer cells and therefore hold promise as phenotypic biomarkers.^23^

Beyond dynamic live-single cell molecular biomarkers, traditional (static) molecular phenotypic biomarkers have been extensively utilized in research to study the effects of genetic alterations and therapeutics in cancer cells. However, their clinical applications have been limited with only a handful in current clinical use.^24, 25^ Examples include circulating proteins in blood/urine for human cancer screening, (e.g., serum PSA for prostate cancer and CA 125 for ovarian cancer), functional proteins in fixed tissue samples for diagnostic and prognostic purposes (e.g., Her2, ER, and PR in breast cancer) and specific circulating proteins to monitor response to treatment and disease progression (e.g., CEA, CA125, CA27.29, PSA, and CA19-9).^26^ Dynamic molecular phenotypic biomarkers remain an untapped resource for cancer diagnosis and risk stratification, as well as have the potential to serve as biomarkers for screening, diagnosis, prognosis, assessment of drug response and in companion diagnostics and disease state monitoring (Fig. 1c). This observation may be due to the modality by which molecular biomarkers are currently being measured.

Importantly, shifting from a fixed/static biomarker molecular measurement to a live/dynamic measurement may indeed provide both the spatial and temporal resolution needed to maximize molecular phenotypic biomarker information. For example, label-free quantification actin polymerization via membrane fluctuations, in that it relates to protrusive forces and motility, may be an important biomarker to characterize metastatic potential of a given cell.

### Cellular phenotypic biomarkers

Similar to molecular phenotypic biomarkers, cellular phenotypic biomarkers may benefit from the incorporation of dynamic data, on a single cell level, as well as a tissue-based level. For example, and as tumorigenesis is a complex biological process that involves alterations in the mechanical properties of cells,^27^ mechano-transduction^28^-the conversion of mechanical forces into biochemical signals^29^-contributes to developmental,^30^ physiological, and pathological processes in cancer progression.^31, 32^ Work is ongoing to apply microfluidic technologies to create tools that can “squeeze” or mechanically stimulate cancer cells in suspension, derived from blood or urine samples, and thereby assess cellular mechanical properties such as contractility and force generation.^33^ Important recent advances in matrix biology, live single-cell imaging, microfluidics and automated image analysis have made it possible to maintain cancer cells ex vivo, and quantify their mechanical or biophysical properties as “label-free” phenotypic biomarkers.^33, 34^

The aforementioned advances have made it possible to measure cellular phenotypes that take multiple snap-shots of important cellular features that are the resultant actions of multiple integrated biochemical signaling pathways, thereby interrogating a single cancer cell’s signal transduction profile to assess if it is normal or pathological. For example, cancer cells have altered migration rates compared to non-cancer cells from the same tissue. Changes in migration velocity can result from underlying cytoskeletal changes such as shortening of F-actin filaments^35^ or from changes in complex biochemical pathways e.g., aberrant androgen signaling in prostate cancer results in altered cell migration.^36^ Cell spreading is another actin-dependent feature related to cellular invasiveness.^37^ Cell spreading is also correlated with altered DNA synthesis, motility, and differentiation.^21^ Cell deformability (or effective stiffness) is another cellular biomarker used to categorize cell populations and is an end result of multiple biochemical and molecular changes.^34^ Data from several epithelial cell carcinomas, including ovarian and breast, suggest that decreased cell stiffness or increased cell deformability is indicative of malignant transformation and may be a useful measure of metastatic potential.^34, 38, 39^ Table 1 lists important live-cell phenotypic biomarkers and their functions.

**Table 1:** List of live cell phenotypic biomarkers and their implication in tumorigenesis

Phenotypic Biomarker	Measure of	Implication	Ref
Cell tortuosity	Shape	O/A	63
Nucleus area and perimeter dynamics	Proliferation	O/A	64
Cell spreading dynamics	Invasion	M/I	65
Cell migration velocity	Motility	M/I	66
Cell stiffness	Growth, migration	M/I + O/A	67
Cell area/perimeter dynamics	Shape & migration	M/I + O/A	68
Retrograde actin flow velocity (RFV)	Motility	M/I + O/A	14

Note: O/A oncogenicity/aggressiveness, M/I metastasis/invasiveness

As many signaling events that regulate oncogenesis and metastasis are governed by dynamic cytoskeletal events that occur on a sub-micron (sub-cellular) spatial scale and a (sub) milli-second temporal scale, the addition of quantifiable live-cell biomarkers on similar spatial and temporal scales may be useful as a powerful addition to the current suite of cellular phenotypic biomarkers towards ameliorating current clinical applications.

### Comparison of conventional, genomic, and phenotypic biomarkers using diagnostic and prognostic applications as an example

Conventional histopathology is the current gold standard for diagnosis of most cancers (e.g., prostate, breast, lung etc.). Based on the qualitative and subjective evaluation of tissue specimens, routine histopathology suffers from variability in diagnosis, suboptimal sensitivities and the inability to distinguish indolent from aggressive cancers. The relative recent adoption of immunolabeling/IHC for a handful of protein biomarkers (like PD-1, OX40, HER2) has improved the predictive power of histopathology by providing information about tumor subtypes, prognostication and even guiding treatment decisions. However, IHC remains a semi-quantitative method at best, is time consuming and relies on limited tissue sampling and the pathologist’s subjective visual interpretation of staining intensity.

In an attempt to make diagnosis and prognosis more standardized and accurate, several diagnostic tests based on quantification of genomic cancer biomarkers have been developed.^40–43^ Genomic tests measure selected genetic loci and can be performed on static/formalin fixed paraffin-embedded tissue samples. Predictions of tumorigenicity (prognostication) are based on the quantitative gene expression data of a selected but small number of genes. Although innovative, genomic marker tests suffer from poor “signal-to-noise” ratio due to the significant tumor heterogeneity from bulk-tumor samples that occur in solid tumors such as prostate and breast cancer.^44–46^ Genetic tests are designed to detect mutational hotspots in cancer-related genes and pose a risk of false negative results when used for testing cancers with unknown mutations.^47^ This is reflective in the relatively low sensitivities (<0.70), or other test performance statistics (like area under the curve or AUC) reported in clinical studies for many of the marketed genomic biomarker tests.^40^

Given the importance of maintaining in vivo cell behavior in vitro and time to culture, traditionally it has been difficult to culture single cell cultures of primary human cells derived from biopsy tissue in vitro for clinical applications. Advances in our understanding of integrin-mediated ECM interactions has enabled rapid culturing of primary human cells (such as prostate) allowing analysis of cells as early as 48 h after sample procurement with the ability to maintain primary cells in vitro for at least 7 days.^3, 48^ A study has shown the use of a well-defined ECM allows for survival and growth of primary prostate cells derived from radical prostatectomy tissue.^3, 48^ Further, the use of a defined ECM environment provides a reference standard to gage and compare how primary cells from different patients interpret a specific and consistent microenvironment. More work in this field is ongoing and shows promise towards the ability to rapidly and reproducibly culture cells derived from patient biopsy.^49^ Additional studies have also been successful at controlling media and ECM conditions, to establish slice cultures, organoid cultures or slow growing (~30 day) single-cell cultures.^1, 2, 49–52^

Live-single-cell cellular and molecular phenotypic biomarkers represent an easily quantifiable result or biomarker representative of a concert of biochemical pathways or the total expression of the complex and inter-related cellular processes that regulate cellular homeostasis and pathogenesis (i.e., genetic (DNA, RNA, RNAi), epigenetic, chromosomal, proteasome, metabolome, etc.). Imaging biophysical biomarkers and monitoring protein expression, sub-cellular localization, and modification states in living cancer cells on a single-cell level may allow direct assessment of cellular function and tumor heterogeneity by quantifying molecular and cellular behavior in individual cells.

Specifically, rapid and quantitative measurement of live-cell phenotypic biomarkers using a microfluidic platform and trained, automatic machine vision, and learning algorithms could provide meaningful, objective information about the invasiveness and growth potential of tumor cells.^53^ Assessment of phenotypic behavior at the resolution of individual cancer cells is advantageous, as it considers functionally important discrete sub-populations of cells that may dictate tumor biology that, otherwise, would be lost in bulk genomic analysis. Furthermore, single-cell analysis allow measurement of the intra-tumor heterogeneity, which itself could be used as a biomarker to predict disease progression and drug resistance.^54^ Moreover, live-single-cell phenotypic biomarkers provide direct measures of functional biological pathways providing vital information such as protein expression, localization or activity. Genomic tests on the other hand do not provide information about protein localization and infer data about protein expression and activity through gene expression (RNA) profiling, which have been shown to have minimal or limited correlations.^55^

Table 2 summarizes the main advantages and disadvantages of the current diagnostic approaches in cancer. A drawback of both genomic and live-single-cell phenotypic diagnostic tests described above, is the loss of spatial information of different cell types relative to each another, tumor microenvironment and to the anatomical features of the tissue. With that, single-cell analysis is buttressed by the ability to assess single-cell behavior when cells interact with a defined ECM or microenvironment. The limitations of most diagnostic tests are a function of the way samples, intended for genotypic or live-single cell phenotypic analysis, are processed for testing and could be a concern in specific cancers like brain or skin malignancies, where tissue anatomy is particularly informative. However, as live-cell diagnostic tests evolve with greater predictive power and improved sensitivities, this may become a minor concern.

**Table 2:** Advantages and disadvantages of conventional, genomic, and phenotypic diagnostic tests

Diagnostic test	Time to results	Categorization of disease state	Tissue sample cellular/molecular classification	Clinical application	Advantages	Disadvantages	Ref
Histopathology	3–7 days	Qualitative broad categorization	Single time point FFPE fixed cell morphology and molecular (protein, mRNA, DNA) classification	Diagnostic	Clinical familiarity	Subjective and variable interpretation	69, 70
					Spatial information	Low sensitivity	
Genomic Tests	14–21 days	Quantitative limited categorization	Single time point FFPE fixed cell no morphology and limited molecular (mRNA & DNA) classification	Screening	High throughput	Poor performance statistics,	71
				Diagnostic	Quantitative	Loss of spatial information	
				Risk stratification	Predictive		
				Therapy selection			
				Monitoring			
Phenotypic tests	1–7 days^a^	Quantitative ^b^composite score predicting severity of disease	Multi-time point live-cell morphology and live-cell/formalin fixed cell molecular (protein, mRNA, DNA) classification on standardized ECM microenvironment	Screening	High throughput	Limited spatial information	34
				Diagnostic	Quantitative	Live-cell assays not yet clinically validated	
				Risk stratification	Predictive	Live-cells not in native microenvironment (^a^live-cells maintained in standardized ECM microenvironment)	
				Therapy selection	Faster results		
				Monitoring			

^a^ projected estimate

^b^ based on preliminary results

There has been a rapid evolution in cancer diagnostics over the last decade.^56^ Cancer diagnostics is moving quickly from reliance on histopathology and immuno-histochemical tissue staining towards the use of quantitative, biomarker-based, personalized approaches (Fig. 1d). In the future, development of diagnostic platforms that incorporate both genomic and phenotypic and biomarkers may allow for improved risk stratification and treatment decision-making.

### Promise of live-cell behavior as a phenotypic biomarker

The expansion in the repertoire of phenotypic biomarkers to include live-cell behavior has the potential to revolutionize cancer diagnostics and drug development in the new era of personalized and precision medicine. Evaluation of live-cell behavior in a rigorously engineered and reproducible environment that maintains consistent cell-cell and cell-matrix interactions will identify new and previously inaccessible phenotypic biomarkers-distinct for cancer and benign cells. Live-cell phenotypic biomarkers can be measured label-free and represent the general characteristics of any malignant cell, irrespective of tumor type.

In addition to diagnosis and prognosis (risk stratification), live-cell phenotypic biomarkers have potential uses in drug screening, companion diagnostics and lead compound discovery.^57^ For example, assessment of cell migration has been employed to screen FDA-approved drugs for effective treatment of metastatic prostate cancer.^58^ The screen was based on the phenotype of cell migration and invasion and depended on assessing the behavior of living prostate cancer cells lines exposed to tested drugs vs. vehicle controls.^58^ Within this same concept, a multi-phenotype assay measuring all live-cell biomarkers outlined in Table 1 could be a potentially powerful tool for compound discovery and FDA approved drug library screening for prostate and other solid tumors.

Indeed, motivated by the need to dissect and interrogate the heterogeneity and multiplicitous nature of many cancers,^59–62^ a number of techniques are emerging that use high throughput microfluidic devices to discern single cell phenotypic behavior often focusing on cellular mechanics such as migration, traction force, cell tensile strength, and extra-cellular matrix / microenvironment cell interaction.^48, 63–73^ Additionally, there are emerging technologies that apply single cell analysis techniques to for improved screening, diagnosis, prognosis, and therapy selection, as well as drug discovery and development that are extensions of more established techniques that use cellular phenotype profiling.^73–75^

Live-cell phenotypic biomarkers could be used as a theranostic (or via a companion diagnostic) approach in which an individual’s live cells could be screened against a panel of chemotherapeutic drugs (singly or in combination) to provide a direct read-out of tumor response, again, given a defined ECM, to drug treatment prior to use in oncology drug trials and clinical practice.^76^ Live-cell phenotypic biomarkers could also be used to design and study the effect of combination therapies and the new class of immune-oncology drugs supporting the potential paradigm shift in cancer diagnosis and treatment in the era of (live-cell) personalized medicine.^77^

General biology is often studied using cellular phenotypic assays as screens to test gene function and compare cancer cells with non-cancer cells as a method to better understand the underpinnings of cancer cell biology.^76^ Using a high-content, automated live cell imaging, phenotypic assay, Pau et al was able to screen cells treated with a small interfering RNA library and identify 2190 genomic targets that disrupted the cell cycle, mitosis, or cell death pathways.^78^ Indeed such large scale screening approaches utilizing live cell phenotypic assays are poised to usher in a novel approach to interrogate cancer cell biology by allowing computer generated models to be developed for studying cancer biology in-silico.^79^

## Conclusions

While much progress has been made in identifying biomarkers in research settings, clinical adoption has been slow. Thus, there remains a significant unmet need for specific and sensitive biomarkers of tumor aggressiveness and metastatic potential that can be used for optimal risk stratification towards treatment decision-making.

Phenotypic biomarkers, particularly those evaluating live-cell behaviors, have the potential to fulfill this need for a quick and actionable biomarker platform to underpin the precision oncology paradigm. The underlying advantage of live-cell phenotypic biomarkers (molecular and cellular) over genomic biomarkers is their universal applicability in multiple solid human tumors, their ability to report on multiple, synchronous biochemical pathways, and their reflection of a cancer cell’s protein expression profile in concert and behavior in its microenvironment. Furthermore, recent technical advances allow for rapid and automated measurement of phenotypic biomarkers in large numbers of live tumor cells enabling big-data analysis at the single cell level to mitigate the challenges of tumor heterogeneity.

Precision oncology stands to benefit enormously from the development of a live-cell phenotypic biomarker (molecular and cellular) platform using high throughput automated approaches. A composite measurement of live-cell phenotypic biomarkers in cancer represents an innovative and actionable addition to personalized cancer diagnosis, risk stratification, and treatment decision-making.^80-88^
